# Contributions of Genetic Evolution to Zika Virus Emergence

**DOI:** 10.3389/fmicb.2021.655065

**Published:** 2021-05-06

**Authors:** Su-Jhen Hung, Sheng-Wen Huang

**Affiliations:** National Mosquito-Borne Diseases Control Research Center, National Health Research Institutes, Tainan, Taiwan

**Keywords:** Zika virus, evolution, virulence, mutation, emergence

## Abstract

Mosquito-borne Zika virus (ZIKV) was considered an obscure virus causing only mild or self-limited symptoms until the explosive outbreaks in French Polynesia in 2013–2014 and in the Americas in 2015–2016, resulting in more than 700,000 cases of the disease, with occasional miscarriage and severe congenital birth defects, such as intrauterine growth restriction, fetal microcephaly, and other neurodevelopmental malformations. In this review, we summarized the evolution of ZIKV from a mundane virus to an epidemic virus. ZIKV has acquired a panel of amino acid substitutions during evolution when the virus spread from Africa, Asia, Pacific, through to the Americas. Robust occurrence of mutations in the evolution of ZIKV has increased its epidemic potential. Here we discussed the contributions of these evolutionary mutations to the enhancement of viral pathogenicity and host-mosquito transmission. We further explored the potential hypotheses for the increase in ZIKV activity in recent decades. Through this review, we also explored the hypotheses for the occurrence of the recent ZIKV epidemics and highlighted the potential roles of various factors including pathogen-, host-, vector-related, and environmental factors, which may have synergistically contributed to the ZIKV epidemics.

## Introduction

Zika virus (ZIKV) belongs to the genus *Flavivirus* together with other important mosquito-borne human viruses, such as dengue virus (DENV), West Nile virus, Japanese encephalitis virus, and yellow fever virus. The flaviviruses have single positive-stranded 11-kb RNA genomes that the 5’ and 3’ untranslated regions flank a polyprotein coding region encoding three structural proteins [capsid, pre/membrane (prM), and envelope (E)] and seven non-structural proteins (NS1, NS2A, NS2B, NS3, NS4A, NS4B, and NS5). The main mosquito vector during epidemics belongs to the genus *Aedes* (*Aedes aegypti* and *A. albopictus*) (Diallo et al., 2014; Guo et al., 2016; Epelboin et al., 2017; Rossi et al., 2018). ZIKV has continuously adapted to mosquitoes and non-human primates in a sylvatic cycle, which then results in a virus reservoir. When ZIKV opportunistically enters the human transmission cycle from the sylvatic cycle, the virus may initiate epidemics in humans. In addition to the *Aedes*-human transmission cycle, non-vectored transmission between humans has been hypothesized because the ZIKV genome can be found in saliva (Musso et al., 2015), urine (Gourinat et al., 2015), and even tears (Miner et al., 2016).

Zika virus was first isolated from non-human primates in the Zika forest of Uganda in 1947 (Dick et al., 1952), and in humans in 1954 (Macnamara, 1954). Since then, it has been continuously isolated in Uganda and Nigeria (Simpson, 1964; Moore et al., 1975), and only sporadic cases with self-limited signs or mild symptoms were reported in Africa (Macnamara, 1954; Simpson, 1964; Moore et al., 1975; Fagbami, 1979) and South-east Asia (Olson and Ksiazek, 1981) until the first major outbreak, evidenced by serology and virology of patients with rash, fever, arthralgia, and conjunctivitis, occurred in Yap Island, Micronesia in 2007 (Duffy et al., 2009). Subsequently, ZIKV resumed low activity, and only a few countries including Cambodia (in 2008, 2009, and 2010), Nigeria (2011), Indonesia (2012), Russia (2012), and the Philippines (2012), reported ZIKV isolations from 2008 to 2012. Afterward, ZIKV caused another larger outbreak in French Polynesia in 2013–2014 (Cao-Lormeau et al., 2014), in which more than 30,000 individuals were observed for suspected infection. During this outbreak, ZIKV infections were first associated with patients with Guillain-Barré Syndrome (GBS), the neuropathic condition characterized by progressive weakness and diminished or absent myotatic reflexes (Walling and Dickson, 2013; Cao-Lormeau et al., 2016). Thereafter, ZIKV spread to several other Pacific islands, including Easter Island, Cook Island, Solomon Islands, New Caledonia, Vanuatu, Tonga, Fuji, Samoa, and American Samoa (Delatorre et al., 2018), and then to South American mainland. In May 2015, Brazil-originating ZIKV dissemination was reported (Zanluca et al., 2015), which then caused an explosive outbreak in September 2015. This outbreak casually linked ZIKV infection and birth defects or disabilities, such as severe microcephaly; the condition was termed congenital Zika syndrome (CZS). Simultaneously, ZIKV quickly spread from Brazil to the Caribbean and Central America and was eventually imported into the United States (Grubaugh et al., 2017). In 2016, at least 175,000 laboratory-confirmed ZIKV cases with more suspected cases from 48 countries and territories were reported in the Americas. Among these ZIKV-infected cases, World Health Organization reported 2,654 cases with CZS by February 2017, and 2,366 cases of these were in Brazil (World Health Organization, 2017). Although the prognosis of microcephaly needs to be more clearly examined, the patients with CZS usually have physical and learning disabilities. Therefore, CZS is a burden to families and societies. During the America epidemics, ZIKV strains continuously spread in Asia and caused an outbreak with 455 confirmed cases in Singapore in 2016 (Ho et al., 2017). Additionally, ZIKV locally disseminated and caused sporadic cases in the Philippines (Lim et al., 2017), Vietnam (Moi et al., 2017), and Thailand (Wongsurawat et al., 2018) in 2015–2016. Microcephaly was still noted among the clinical presentations of patients in Vietnam and Thailand, which indicated the general occurrence of CZS in America and Asia after 2015. Nonetheless, the reasons why ZIKV cases with CZS were not identified before 2015 is still unclear.

One of the plausible hypotheses is that ZIKV acquired some virulence mutations which contributed to the neurological infection in human fetal brains (Table 1). The nucleotide changes, along with amino acid substitutions, continuously accumulated over time and in different geographies, resulting in ZIKV evolution (Liu et al., 2019). The accumulated genetic diversity in the ZIKV population may increase the potential to cause severe diseases. To expand on this issue, we reviewed ZIKV evolution from the first isolation to recent emergent outbreaks and discussed the recent evidence on how the genetic changes affected ZIKV phenotypes, and on why ZIKV has become an emerging pathogen in recent outbreaks.

**TABLE 1:** Potential factors contributing to the recent emergence of Zika virus (ZIKV).

**Potential factors**		**Description**	**References**
***Pathogen***			
Mutation	prM-S139N	prM-S139N mutant causes a more severe microcephalic phenotype with a thinner cortex, more robust brain cell apoptosis, and more NPC differentiation disruption in mice	Yuan et al., 2017
Mutation	NS1-A982V	NS1-A982V mutation enhances ZIKV transmission in a mosquito-mouse-mosquito transmission cycle	Liu et al., 2017
		NS1-A982V mutation of ZIKV enhances the inhibition of interferon-beta production	Xia et al., 2018
Mutation	E-V763M	E-V763M mutation increases ZIKV replication, neurovirulence in neonatal mice, and maternal-to-fetal transmission	Shan et al., 2020
Mutation	C-T106A, prM-V123A, NS1-A982V, and NS5-M3392V	ZIKV with C-T106A, prM-V123A, NS1-A982V, and NS5-M3392V mutations has a fitness advantage	Liu et al., 2021
***Host***			
Genetics	Host genome background	The pathogenesis of discordant and dizygotic twins from ZIKV-infected mothers was compared, and host genetics was found to substantially affect the severity of a ZIKV infection, even when infected with the same strain	Caires-Junior et al., 2018
Immunity	Preexisting anti-flavivirus immunity	Previous DENV immunity had no or cross-protection impact against ZIKV infection	Gordon et al., 2019; Rodriguez-Barraquer et al., 2019; Carvalho et al., 2020
***Environment***			
Temperature	Climate change	Elevated temperatures can expand the geographic vector range, decrease the extrinsic incubation period of the pathogen, and increase the female mosquito biting rate	Morin et al., 2013; Paz and Semenza, 2016

*prM-S139N, S139N mutation in the pre-membrane (prM) coding region; NS1-A982V, A982V mutation in the non-structural protein 1 (NS1) coding region; E-V763M, V763M mutation in the envelope (E) coding region; C-T106A, T106A mutation in the capsid (C) coding region; prM-V123A, V123A mutation in the pre-membrane (prM) coding region; NS5-M3392V, M3392V mutation in the non-structural protein 5 (NS5) coding region; DENV, Dengue virus; ZIKV, Zika virus.*

## ZIKV Genetic Evolution: Genetic Changes From Obscureness to a Prominent Emerging Pathogen

**FIGURE 1:**
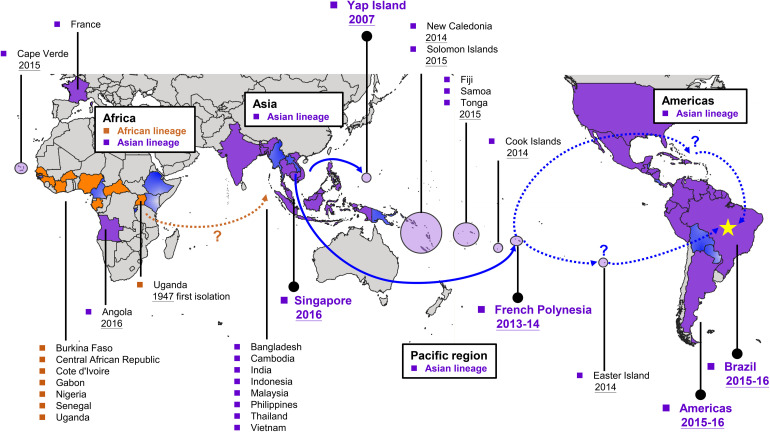
Global dissemination of Zika virus (ZIKV). ZIKV originated from Africa and was then introduced to Asia. Thereafter, ZIKV continuously evolved in Asia and began to cause outbreaks in the Pacific region from 2007. The 2013–2014 French Polynesia strain became the common ancestor worldwide and resulted in recent ZIKV outbreaks in the Americas. According to phylogenetic analysis, the African lineage viruses were circulated mainly in African countries, whereas those circulated in Asia, the Pacific regions, and the Americas were Asian lineage viruses. Countries or areas that have locally acquired ZIKV cases are colored with orange (African lineage circulation), purple (Asian lineage circulation), or gradient blue (the circulating lineage has not been confirmed).

The first ZIKV phylogenetic study was conducted after the 2007 Yap outbreak (Lanciotti et al., 2008). According to a phylogenetic study of the complete open reading frame sequence of the polyprotein, ZIKV has two lineages, the African (Nigeria, Senegal, and Uganda strains) and Asian (Malaysia 1966, Yap 2007, and Cambodia 2010) lineages (Haddow et al., 2012). According to the timescale of a phylogenetic tree estimation of ZIKV evolution (Delatorre et al., 2018), Asian ZIKV strains had two independent disseminations from Southeast Asia into the Pacific region–the first in Yap Island and the second in French Polynesia (Figure 1). With regards to the evidence that the Cambodia 2010 strain is the closest to the French Polynesia 2013 ZIKV outbreak strain, the former was sequentially introduced into the Pacific region and resulted in the 2013–2014 French Polynesia outbreak (Figure 1; Cao-Lormeau et al., 2014). Later, all of the Asian lineage strains isolated from American countries from 2014 including Brazil (Campos et al., 2015; Zanluca et al., 2015; Calvet et al., 2016), Colombia (Camacho et al., 2016), Puerto Rico (Lanciotti et al., 2016), and Guatemala (Lanciotti et al., 2016) showed phylogenetic and temporal closeness in the phylogenetic tree with >99% nucleotide identity with the French Polynesian strains (Lanciotti et al., 2016). Therefore, French Polynesian strains were suggested to simultaneously disseminate into the Easter Island, New Caledonia, the Cook Islands, and the Americas at approximately the same time, suggesting that these strains spread to the Americas and then caused a ZIKV epidemic globally in 2015–2016 (Figure 1). Simultaneously, the French Polynesian strain continued its dissemination in the Pacific countries including Samoa, Fiji, Tonga, and American Samoa in a stepping-stone process in 2015–2016 (Delatorre et al., 2018). Taken together, phylogenic evidence of ZIKV showed that the virus was first isolated in Africa and then was found in Asia. Afterward, ZIKV continuously evolved in Asia. ZIKV began to cause epidemics in the Pacific from 2000, including the 2007 Yap outbreak and the French Polynesia 2013–2014 outbreak. French Polynesia was suggested as the main origin for global ZIKV dissemination, which was sequentially introduced into the Americas where thousands of CZS cases were reported.

## Contribution of ZIKV Mutations to Fetal Microcephaly and Mosquito Transmission

To date, severe CZS cases have been found to be primarily caused by Asian ZIKV strains (D’Ortenzio et al., 2016), raising an important scientific question. That is, whether the accumulated mutations in ZIKV, particularly those acquired when the African strain evolved to the Asian lineages, contributes to the increase in CZS severity or transmissibility in mosquito vectors. Thus, several studies have investigated the phenotypic differences between African and Asian strains; however, the results obtained did not agree with the intuitive hypotheses. *In vitro* tissue culture evidence revealed that the African strains exhibit higher growth rates and induce higher rates of cellular apoptosis than the Asian strains in primate cell lines, such as Vero, human embryonic kidney (HEK293) cells, human neuronal progenitor cells (NPCs) as well as neuroblastoma, glioblastoma, and monocyte-derived dendritic cells (Simonin et al., 2016; Anfasa et al., 2017; Bowen et al., 2017; Shao et al., 2017; Sheridan et al., 2017; Smith et al., 2018). Similarly, an *in vivo* model conferred that the African strains have higher virulence than the Asian strains in mice (Dowall et al., 2017; Shao et al., 2017). Moreover, mosquitoes are more susceptible to the African strains than the Asian strains (Weger-Lucarelli et al., 2016; Azar et al., 2017; Roundy et al., 2017). Cumulatively these studies demonstrate that the Asian strains, responsible for the ZIKV outbreaks in the Pacific and the Americas, were less virulent with lower vector competence than the African strains.

Due to this evidence that was counterintuitive to the hypothesis for ZIKV emergence and the epidemic trends of ZIKV after 2007, more recent studies have focused on phenotypic comparison of the ancestral (Cambodia 2010) strain and different contemporary Asian strains isolated after 2010 (Liu et al., 2017; Yuan et al., 2017). One study utilized a mouse embryonic microcephaly model to compare the virulence of the ancestral and contemporary strains (Yuan et al., 2017). *In vitro* NPC results indicated that contemporary Asian strains showed a substantial increase in viral growth and cellular apoptosis in contrast with the ancestral strain. Contemporary strains also exhibited higher virulence in mouse brain tissue, and sequentially induced severe microcephaly phenotypes in embryonic brains *in vivo* (Liu et al., 2017). The authors compared the transmissibility of the ancestral Cambodia strain and contemporary Asian strain between the interferon receptor-deficient adult mice and *A. aegypti* mosquitoes. Although the ancestral and contemporary strains exhibited similar growth rates in AG6 mice, the contemporary China 2016 strain not only enhanced the ZIKV NS1 viremia, but also exhibited higher infectivity rates in the mosquitoes feeding on ZIKV-infected AG6 mice. In addition, the contemporary strain exhibited a fitness advantage in contrast with the ancestral strain, in both mice and mosquitoes (Liu et al., 2021). Taken together, the contemporary strain exhibits higher neurovirulence, causing microcephaly, as seen in the mouse model, enhancing the viral infectivity in mosquitoes, and possessing superior fitness than the ancestral strain *in vivo*.

Considering the numerous evolutionary mutations that have been identified in the contemporary strains, via phylogenetic analysis, various studies have applied reverse genetics systems to assess the contributions made by these mutations of ZIKV phenotype (Table 1; Liu et al., 2017; Yuan et al., 2017; Shan et al., 2020; Liu et al., 2021). Results have shown that the prM-S139N mutation accelerates viral replication in human NPCs, as well as viral virulence in the neonatal mouse model (Yuan et al., 2017). Furthermore, prM-S139N mutants have a more severe microcephalic phenotype with a thinner cortex, more robust brain cell apoptosis, and more NPC differentiation disruption, in contrast with the wild-type virus. Meanwhile, the E-V763M mutation was found to increase ZIKV replication, neurovirulence in neonatal mice, and maternal-to-fetal transmission (Shan et al., 2020). Additionally, the NS1-A982V mutation enhanced ZIKV infectivity in *A. aegypti* mosquitoes and elevated NS1 antigenemia without altering infective virus propagation in AG6 mice (Liu et al., 2017). Nonetheless, the NS1-A982V mutation enhanced ZIKV transmission not only from AG6 mice to mosquitoes, but also in a mosquito-mouse-mosquito transmission cycle. Additionally, the NS1-A982V mutation caused the NS1 protein to bind to TBK1, and subsequently reduce its phosphorylation level, which may inhibit interferon-beta production (Xia et al., 2018). In addition to single mutation, contemporary Asian strains possess C-T106A, prM-V123A, NS1-A982V, and NS5-M3392V, which serve to synergistically increase viral fitness in mice and mosquitoes (Liu et al., 2021). Cumulatively, these findings indicate that not only several single mutations, but also multiple mutations, have altered ZIKV phenotypic characteristics, including disease severity, transmission, and viral fitness.

## Non-Mutation Based Potential Factors Responsible for the Recent ZIKV Emergence

Although additional ZIKV mutations, responsible for enhancing ZIKV neurovirulence, mosquito infectivity, and fitness, are likely to be characterized in the future, the general consensus is that other factors, aside from genetic mutations, have also contributed to the large outbreaks that occurred in 2015–2016 in the Americas (Table 1). Due to the nature of error-prone RNA virus replication, mutations can readily occur during ZIKV evolution and may represent a founder’s effect with a neutral fitness. Meanwhile, considering that the African strains are more virulent than the Asian strains, an alternative hypothesis is that all ZIKV strains have the rare capacity to cause severe neurological diseases through host-vector (urban), or host-host (sexual) transmission cycles (Rossi et al., 2018). Therefore, severe cases can only be detected by public health surveillance systems in a large outbreak; whereas in small outbreaks, such as the 2007 Yap State outbreak with approximately 5,000 human cases, the severe cases were not prevalent enough to be sufficiently recognized (Duffy et al., 2009). In contrast, with more than 30,000 cases of ZIKV diagnosed in the French Polynesia, and even greater numbers in the Americas, substantial rates of GBS or CZS cases were also detected during these devastating outbreaks. This alternative hypothesis may explain why infection by both African and Asian strains can result in fetus microcephaly and mosquito infectivity elevation. However, this hypothesis does not address one key question. That is, why was ZIKV activity substantially increased after the French Polynesia outbreak and robustly elevated during the American outbreak?

In addition to the pathogen, host factors including genetics and immunity represent major components in the epidemiologic triad of infectious diseases (Table 1). In a study comparing the NPCs from three pairs of dizygotic twins with different CZS in ZIKV infection, the cells exhibited diverse gene profiles for major regulators of the neurodevelopmental program, including the mTOR and WNT pathways. Moreover, the NPCs from affected individuals were more susceptible to ZIKV infection with accelerated viral replication and reduced cell growth (Caires-Junior et al., 2018). Hence, host genetics substantially affect the severity of ZIKV infection, even for dizygotic twins infected with the same strain *in utero*. Nonetheless, no specific loci associated with the CZS has been identified, suggesting that CZS may be a multifactorial disease with oligogenic and/or epigenetic mechanisms. Despite host genetics, pre-existing flavivirus immunity was suggested to modulate subsequential ZIKV disease outcome. Early *in vitro* and mouse studies reported that preexisting anti-DENV immunity associated with ZIKV pathogenesis via antibody-dependent enhancement (ADE) (Stettler et al., 2016; Bardina et al., 2017; Rathore et al., 2019); however, clinical and epidemiological evidence for ADE has not been provided yet. In contrast, human studies demonstrated that previous DENV immunity had no or cross-protection impact against ZIKV infection (Halai et al., 2017; Moreira-Soto et al., 2017; Castanha et al., 2019; Pedroso et al., 2019; Katzelnick et al., 2020; Michlmayr et al., 2020; Tonnerre et al., 2020; Petzold et al., 2021). In the Nicaraguan and Brazil pediatric cohorts, prior DENV infection and pre-existing anti-NS1 DENV antibodies were associated with reduced risk of ZIKV infection and disease (Gordon et al., 2019; Rodriguez-Barraquer et al., 2019). One epidemiological study observed that the areas with large DENV epidemics within 6 years had lower rates of CZS, which suggesting the protection role of recent DENV exposure (Carvalho et al., 2020). In murine models, DENV-immune *Ifnar1^–/–^* mice displayed that DENV-reactive CD8+ T cells mediate cross-protection against subsequent ZIKV challenge (Wen et al., 2017a, b; Regla-Nava et al., 2018). Heterotypic cross-protection may drive ZIKV evolution under the selection of flavivirus-reactive CD8+ T cell immunity in Asia where ZIKV has been circulating for decades in the face of heterotypic flavivirus immunity. In addition, heterotypic cross-protection provides potential explanation why only few CZS patients have been reported in Africa, another potential ZIKV-circulating continent with a high prevalence of heterotypic flaviviruses (Amarasinghe et al., 2011; Lourenco et al., 2018; Hill et al., 2019). Nonetheless, one aspect that cannot be discounted is the possibility that the number of ZIKV cases are underestimated in certain African nations due to potential misdiagnosis of ZIKV infection in areas endemic for other similar diseases, such as dengue fever.

The environment accounts for the third major component in the epidemiologic triad (Table 1). The 2015 El Niño resulted in record-breaking global temperatures, accompanied by severe drought throughout northern and eastern South America, during the second half of the year. These elevated temperatures did not only decrease the extrinsic incubation period, but also expanded the host range and increased the female mosquito biting rate (Morin et al., 2013; Paz and Semenza, 2016). In addition, the expansion of the *Aedes spp.* mosquito range correlates with an increase in water storage during regional periods of drought (Pontes et al., 2000). According to the correlation that was made regarding climate change and *Aedes spp.* expansion, a model was recently developed and applied to predict the time required for each *A. aegypti* life cycle based on the biophysical response to environment conditions (Iwamura et al., 2020). Results demonstrated that the world became approximately 1.5% more suitable per decade for the development of *A. aegypti* from 1950 to 2000 and will accelerate to 3.2–4.4% by 2050, emphasizing the recent gradual expansion of *A. aegypti*. These studies simultaneously indicate that global climate change, including temperature elevation, potentially increase the *Aedes* spp. development and expansion. Hence, all three major components, namely, pathogen, host, and environment, likely contribute to the observed increase in ZIKV prevalence and, as such, must each be taken into account when considering the recent ZIKV emergence.

## Conclusion

We believe that multiple components have likely synergistically contributed to the debilitating ZIKV outbreaks observed in recent years. Although the number of ZIKV cases reported globally has declined since 2017, surveillance data shows that ZIKV continues to be detected in several areas and in travelers from endemic regions. In future, global surveillance, differential diagnosis, vector control, and virus sequencing are required for prompt monitoring of the transmission dynamics and providing early warnings of possible ZIKV outbreaks in future. Moreover, ZIKV vaccine development is urgently needed to control the next wave of a potential outbreak; however, preexisting flavivirus antibodies in people with prior flavivirus infection or vaccination call for great caution in the design and implementation of vaccines.

## Author Contributions

S-WH and S-JH: conceptualization, data curation, writing – original draft, and writing – review and editing. S-WH: project administration, resources, supervision, and visualization. Both authors contributed to the article and approved the submitted version.

## Conflict of Interest

The authors declare that the research was conducted in the absence of any commercial or financial relationships that could be construed as a potential conflict of interest.
